# Imprecise perception of hand position during early motor adaptation

**DOI:** 10.1152/jn.00447.2023

**Published:** 2024-05-08

**Authors:** Matthias Will, Max-Philipp Stenner

**Affiliations:** ^1^Department of Neurology, Otto-von-Guericke University, Magdeburg, Germany; ^2^Department of Behavioral Neurology, Leibniz Institute for Neurobiology, Magdeburg, Germany; ^3^Center for Behavioral Brain Sciences, Magdeburg, Germany; ^4^Center for Intervention and Research on adaptive and maladaptive brain Circuits underlying mental health (CIRC), Jena-Magdeburg-Halle, Germany

**Keywords:** cognitive strategy, intermodal attention, kinesthesia, position sense, visuomotor adaptation

## Abstract

Localizing one’s body parts is important for movement control and motor learning. Recent studies have shown that the precision with which people localize their hand places constraints on motor adaptation. Although these studies have assumed that hand localization remains equally precise across learning, we show that precision decreases rapidly during early motor learning. In three experiments, healthy young participants (*n* = 92) repeatedly adapted to a 45° visuomotor rotation for a cycle of two to four reaches, followed by a cycle of two to four reaches with veridical feedback. Participants either used an aiming strategy that fully compensated for the rotation (*experiment 1*), or always aimed directly at the target, so that adaptation was implicit (*experiment 2*). We omitted visual feedback for the last reach of each cycle, after which participants localized their unseen hand. We observed an increase in the variability of angular localization errors when subjects used a strategy to counter the visuomotor rotation (*experiment 1*). This decrease in precision was less pronounced in the absence of reaiming (*experiment 2*), and when subjects knew that they would have to localize their hand on the upcoming trial, and could thus focus on hand position (*experiment 3*). We propose that strategic reaiming decreases the precision of perceived hand position, possibly due to attention to vision rather than proprioception. We discuss how these dynamics in precision during early motor learning could impact on motor control and shape the interplay between implicit and strategy-based motor adaptation.

**NEW & NOTEWORTHY** Recent studies indicate that the precision with which people localize their hand limits implicit visuomotor learning. We found that localization precision is not static, but decreases early during learning. This decrease is pronounced when people apply a reaiming strategy to compensate for a visuomotor perturbation and is partly resistant to allocation of attention to the hand. We propose that these dynamics in position sense during learning may influence how implicit and strategy-based motor adaption interact.

## Graphical abstract

**Figure:**
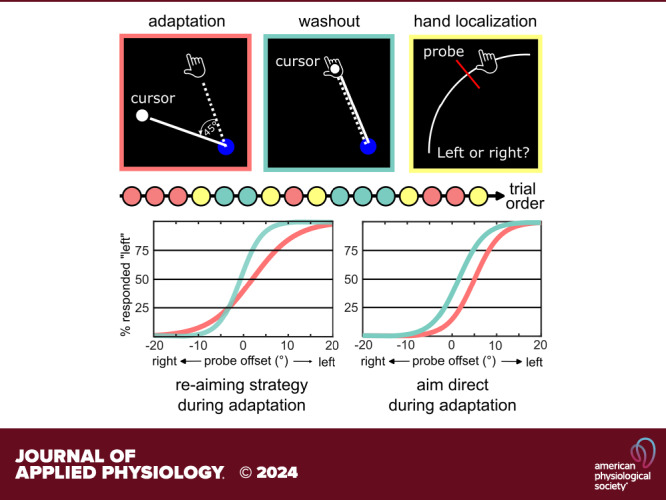

## INTRODUCTION

Accurate and precise perception of limb position is essential for movement control (1). In recent years, the role of position sense for the flexibility of movements has attracted considerable attention, specifically its role for implicit motor adaptation (2). Implicit adaptation is an automatic, nonvoluntary process (3, 4), as opposed to explicit or strategy-based motor adaptation, which involves deliberation and cognitive effort (5, 6). Recent studies have linked implicit adaptation of arm movements to shifts in hand perception and to the precision with which people localize their hand (7, 8). Specifically, the asymptote of implicit adaptation, i.e., the maximum possible adaptation, depends on the precision, or inverse variability, of proprioception. However, previous studies have assumed that this precision remains static across learning. Here, we examined whether the variability of perceived hand position, as a proxy for precision, actually changes during early motor adaptation.

One reason why proprioceptive precision could change early during adaptation is a shift in attention. In a multisensory environment, such as during visuomotor adaptation, attention can increase the precision of information in a certain sensory modality over information from other modalities (9). During visuomotor adaptation, it is plausible that attention shifts from proprioception to vision, in particular, when subjects use a cognitive reaiming strategy to compensate for visual error. Indeed, strategy-based learning is associated with eye movements toward aim points as an indicator of shifted visuospatial attention (10, 11). We thus hypothesized that a reaiming strategy during visuomotor adaptation deemphasizes proprioceptive feedback, and thereby reduces the precision of hand localization. We expected this reduction early during motor adaptation, when cognitive strategies influence adaptation most strongly (6).

Hand localization reports represent a combination of information from various sources, including vision, efferent information, and proprioception. Imprecise proprioception should result in less precise, i.e., more variable, hand localization reports. We thus investigated whether the variability in hand localization increases at the beginning of visuomotor adaptation, and how such an increase is related to the use of a cognitive strategy.

Our experimental design alternated short movement cycles of visuomotor adaptation and washout, allowing us to repeatedly probe hand localization at an early stage of motor learning and estimate proprioceptive precision by computing variance in perceived hand position across repetitions. To ensure robustness of our results, we used two complementary methods for hand localization. To test how precision changes in relation to implicit and strategy-based learning, we examined hand localization during a combination of implicit and strategy-based learning (*experiment 1*) and under conditions of purely implicit learning (*experiment 2*). In a third experiment, we manipulated attention directly. Our results confirm that hand localization becomes less precise during early motor adaptation. We find that this decrease in precision occurs rapidly once a visuomotor rotation is introduced, that it is most pronounced in the presence of a reaiming strategy (*experiment 1*) and less pronounced when reaiming is abolished (*experiment 2*) or when participants can focus on hand localization (*experiment 3*).

## MATERIALS AND METHODS

### Subjects

Ninety-two healthy, young volunteers took part in three experiments. Thirty participated in *experiment 1* (10 females, average age 26 yr, range 20–33 yr), 33 in *experiment 2* (11 females, average age 25 yr, range 20–31 yr), and 29 in *experiment 3* (13 females, average age 26 yr, range 22–33 yr). These cohort sizes were determined based on extensive pilot testing. Participants were recruited via local participant databases and from staff and students of Otto-von-Guericke University and the Leibniz Institute for Neurobiology. They received a monetary reimbursement. All participants were right-hand dominant, verified by the Edinburgh handedness test, and had normal or corrected-to-normal vision. They gave written informed consent to the study protocol before participation. The study was approved by the ethics committee at the university hospital Magdeburg, and conducted in accordance to the Declaration of Helsinki.

### Apparatus

Participants moved their right arm while holding on to the handle of a two-link robotic manipulandum (Kinarm end point laboratory, BKIN technologies). Vision of both hands and arms was occluded by a semisilvered mirror, and a black cloth draped over their shoulders and arms. The mirror provided visual feedback from an LCD monitor (LG47LD452C, LG Electronics, 47 in., 1,920 × 1,080 pixel resolution) mounted above, whose display was facing downward. Participants were seated in a comfortable chair with their forehead resting against a soft leather patch at the height of the monitor. Kinematic data were recorded at a sampling rate of 1,000 Hz, and with a spatial resolution of 0.1 mm. In some experimental conditions, participants used a foot pedal for additional responses. The experiment was conducted in a dimly lit and quiet room.

### General Experimental Design

**Figure 1.**
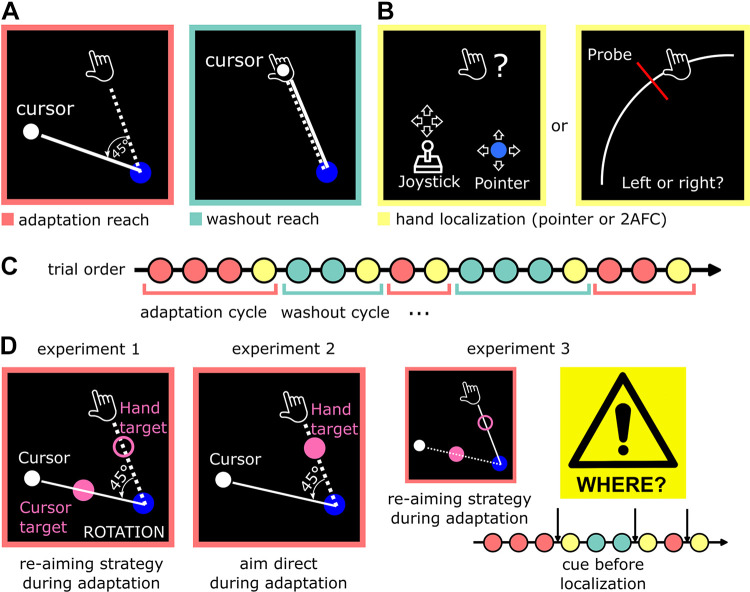
Experimental methods. *A*: participants performed reaching movements, during which visual feedback was either 45° rotated relative to the actual hand position (adaptation reach, red), or aligned with the actual hand position (washout reach, cyan). *B*: after some reaches, performed without visual feedback, participants were asked to report their perceived hand position via one of two methods. They either moved a pointer to the location at which they perceived their right hand, using the left handle of the robotic manipulandum similarly to a joystick (*left*), or judged their right-hand position relative to a visual probe stimulus (*right*). *C*: schematic display of the trial order, in which short adaptation and washout cycles repeatedly alternated. Each cycle consisted of one, two, or three consecutive adaptation or washout reaches and ended with a hand localization trial (yellow). *D*: participants had to move from a starting position (blue circle) through a target. In *experiments 1* (*left*) and *3* (*right*), two targets were displayed (pink ring and dot). During adaptation cycles, participants were instructed to use a compensatory movement strategy to make the cursor slice through the cursor target. In washout cycles (not shown), the cursor and hand target were identical. In *experiment 2* (*middle*), only one target was displayed, and participants were instructed to aim directly at the target during both adaptation and washout cycles. In *experiment 3*, a cue informed participants about an upcoming localization before performing a reaching movement, so they could prepare for hand localization, and focus on their hand.

The goal of this study was to examine the precision of hand localization during the early stage of adaptation to a visuomotor rotation. Perceptual precision can be estimated from the variability of psychophysical reports, the computation of which requires a large number of repetitions. Our interest, however, was in the first few trials of adaptation, when strategy-based learning is most pronounced (6). To capture the expected transient effects on perception, and nevertheless have sufficient repetitions for computing variability, our paradigm required participants to repeatedly adapt and deadapt for short cycles of a few movements each. In each cycle, they reported their perceived hand position once, following the last movement of that cycle (as shown in Fig. 1*C*).

### Reaching Task

Participants produced centre-out reaching movements with their dominant right arm in a horizontal plane, “slicing” through a visual target. Before each reach, the participants’ right hand was passively moved by the robotic manipulandum to the start position (blue circle with radius of 0.5 cm, 15 cm to the right from body midline, 15 cm from the front edge of the table). After keeping their hand inside the home position for 500 ms, the targets appeared at a distance of 10 cm from the home position. In *experiments 1* and *3*, two targets were displayed, a cursor target (pink unfilled circle in Fig. 1*D*, radius of 0.5 cm) and a hand target (pink filled circle, radius of 0.5 cm). In *experiment 2*, a single target (pink filled circle, radius of 0.5 cm) was displayed. Participants were instructed to produce rapid, straight movements and to stop shortly behind the target. There was no time limit for the initiation of the movement. However, once the movement was initiated, participants had to cross the target radius within 300 ms. Movement onset was defined as the point in time when a movement exceeded a velocity threshold of 5 cm/s, and a radial distance from the home position of 0.5 cm. If the time to cross the target radius exceeded 300 ms, they saw an error message on the display (“TOO SLOW”) and heard an error sound. After detection of movement offset (defined as velocity falling below a threshold of 5 cm/s), their hand was locked in end point position for 500 ms, and the trial ended.

Participants saw a cursor (white dot of 0.3 cm radius) as online visual feedback of the position of their right hand during active hand movements. The cursor was not shown during passive return movements. During adaptation cycles (red-outlined panel in Fig. 1*A*), cursor movement was rotated 45° relative to the actual movement trajectory while cursor and actual hand position were congruent during washout cycles (cyan-outlined panel in Fig. 1*A*). Depending on the experiment, the rotation was either consistently 45° in clockwise (CW) direction, or alternated between clockwise (CW) and counterclockwise (CCW) directions in different cycles. Adaptation and washout cycles could consist of two, three, or four consecutive reaches, determined pseudorandomly (equal number of cycles of each length). We varied the number of reaches per cycle to reduce predictability of the localization task, which concluded each cycle.

### Hand Localization Task

A single hand localization trial, as illustrated in the yellow-outline panels in Fig. 1*B*, concluded each cycle. These trials began much like the reach trials described in section *Reaching Task*. In *experiments 1* and *2*, the cursor was shown while the hand was held still in the home position. Upon movement onset, all visible stimuli disappeared, including the cursor and targets. In *experiment 3*, the cursor was not shown during hand localization at all. In all experiments, the right hand was held in the end point position by the robotic manipulandum until participants indicated their perceived hand position, and participants were instructed not to try to move the hand while it was locked in place. Across experiments, we used one of two different hand localization methods, as follows.

#### Pointer method.

For hand localizations using the pointer method (Fig. 1*B,* *left*), a pointer (blue dot, radius 0.3 cm) appeared upon movement offset in a random location along an invisible, horizontal line in front of the participant (ranging from 5–21 cm to the right of the body midline, 10 cm from the front edge of the table). Participants had to move the pointer to the position where they perceived their right hand. They used their left hand to control the pointer by applying isometric force to the left robotic arm’s handle, which functioned similarly to a joystick. Once participants had aligned the pointer with their perceived right-hand position, they pressed a pedal with their right foot to submit the pointer position. For each reported hand position, a graded performance score was calculated based on the distance between the pointer and the actual hand position (100 points for a distance of 0 cm, 0 points for a distance of 10 cm or more). Scores were summed across 36 adaptation and washout cycles (forming one experimental block) to obtain a total performance score for that block, which was displayed at the end of each block to motivate accurate hand position reports.

#### Two-alternative forced choice method.

For hand localizations using the two-alternative forced choice (2AFC) localization method (Fig. 1*B,* *right*), participants had to judge the position of a visual probe stimulus relative to the position of their right hand. Upon movement offset, a white circular arc was displayed, spanning 140° (from −25° to 115° CCW relative to midline), at a radius equal to movement distance. The actual right-hand end point position was somewhere along the arc. A red tick mark was displayed on the arc at an angular offset of ±3.33°, ±10°, or ±20° relative to the actual hand position (in pseudorandom order). Participants had to report if the probe was presented to the left or right relative to their right hand by pressing either a left or right foot pedal with their corresponding foot. If they answered correctly, a point was added to their overall performance score for each block (of 36 trials), which was revealed to participants at the end of each experimental block.

### *Experiment 1*: Reaiming Task

The goal of the first experiment was to examine if precision of perceived hand position decreases during the early stage of visuomotor adaptation. Specifically, participants performed the reaching task with short alternating cycles of adaptation and washout, as described above. During adaptation cycles, cursor movement was rotated 45° CW or CCW. Two targets were displayed (20° and 65° CCW relative to the midline, ±7.5° jitter between cycles). Participants were instructed to move the cursor through the cursor target (filled pink circle) by aiming for the hand target (unfilled pink circle) during adaptation cycles, i.e., to use an aiming strategy (see Fig. 1*D,* *left*). As the angle between both targets corresponded exactly to the angle of visuomotor rotation, the instructed strategy could perfectly compensate for the rotation (4). During washout cycles, cursor and hand position were aligned, and participants were instructed to move the cursor through the filled circle, while the unfilled circle was task-irrelevant. Visuomotor rotation direction, and the location of the filled and the unfilled circle, were reversed from one adaptation cycle to the next, so that participants needed to move to different targets in consecutive adaptation cycles. Thus, a rotation in CW rotation was associated with the instruction to aim for the target at 65° (bringing the cursor to the target at 20°), while a rotation in CCW rotation was associated with the instruction to aim for the target at 20° (bringing the cursor to the target at 65°). The required movement direction during a washout cycle was identical to the required movement direction in the preceding adaptation cycle. A text below the start position indicated whether subjects were in an adaptation cycle (“ROTATION”) so they could apply the instructed reaiming strategy.

Participants were assigned to two groups, who indicated perceived hand position using different hand localization methods (pointer group and 2AFC group, each *n* = 15). Both groups performed multiple blocks of the task, with each block consisting of 18 adaptation cycles alternating with 18 washout cycles (108 reaches and 36 localizations in total per block, circa 12 min per block). Between blocks, participants took a short break (2 min). The pointer group performed one baseline block, consisting of 36 cycles with aligned cursor movement, followed by six blocks of alternating adaptation and washout cycles (18 cycles each). The number of blocks was chosen to allow for sufficient trial numbers to compute the shift and variability of hand localization for the second, third, and fourth reach in a cycle separately. Given that we observed no significant differences in localization variability between baseline and washout, we did not include a baseline block for the 2AFC group. Instead, the 2AFC group completed seven blocks of alternating adaptation and washout.

### *Experiment 2*: Aim-Direct Task

In the second experiment, we tested if the reduction in hand localization precision during early visuomotor adaptation observed in *experiment 1* depended on the use of a reaiming strategy. Participants were instructed to ignore the visuomotor rotation during adaptation cycles and aim directly at the target (see Fig. 1*D,* *middle*). This is a widely used method to exclude strategic aiming (12, 13). As visual landmarks can promote the use of compensatory movement strategies (14), we presented only one target (at 45° relative to midline). As there was only one target in this task, target jitter was expanded to ±15° to ensure a broad range of movements. Furthermore, visuomotor rotation was limited to CCW direction in *experiment 2*. Again, participants were assigned to two groups, who reported perceived hand positions using different hand localization methods (pointer group, *n* = 15, or 2AFC group, *n* = 18). As in *experiment 1*, the experiment consisted of multiple blocks, each containing 18 adaptation and 18 washout cycles, in alternating order. The pointer group performed six such blocks, whereas the 2AFC group completed seven blocks (three participants of the 2AFC group completed only six blocks).

### *Experiment 3*: Cued Localization

To investigate the influence of attention on the reduction of hand localization precision, we conducted a third experiment, in which we increased the predictability of localization trials by cueing. Cueing is an established means to direct attention (e.g., Ref. 15). During reaching, participants performed a reaiming task as in the first experiment. The visuomotor rotation was 45° in CCW direction. The hand target was always at 20° (±15° target jitter) relative to midline (cursor target 45° relative to hand target). In contrast to *experiments 1* and *2*, where the exact time point of a localization was unknown, participants in *experiment 3* were explicitly informed beforehand whether a reach would be followed by the hand localization task. This was done by presenting a large warning symbol along with a text (“WHERE?”) on the screen in the beginning of localization trials (see Fig. 1*D,* *right*). In localization trials, participants could therefore focus attention on the position of their hand during the reaching movement. In one group, two targets were presented at the beginning of localization trials (up to the time of movement onset; two-target group, *n* = 15), and participants had to move their unseen hand through the hand target. In the other group, two targets were present for all reaches that did not require localization, whereas localization trials had only a single target. Given that all other trials had two targets, this additionally signaled the upcoming localization trial, and further minimized any attention away from the hand (one-target group, *n* = 14). In localization trials in both groups, all visual stimuli, including targets, disappeared upon movement onset, like in *experiments 1* and *2*. In both groups, perceived hand position was reported using the pointer method. Participants performed three experimental blocks, as described above, resulting in 54 hand position reports for adaptation and washout, respectively.

### Data Analysis

Data were analyzed in MATLAB R2020b (The MathWorks Inc.). We recorded hand position throughout the experiment and computed movement direction at maximum velocity, movement extent, and movement curvature offline, based on hand position data for the outward movement. For *experiment 1*, we defined movement direction as positive in the direction of expected adaptation, i.e., in CW direction for the target associated with CCW rotation, and in CCW direction for the target associated with CW rotation. For *experiments 2* and *3*, movement direction was always defined to be positive in the CW direction, as rotation direction was always CCW. Movement extent corresponded to the radial distance from the home position to the movement endpoint. Movement curvature was computed via the linearity index (LI), which was defined as the ratio between the maximum perpendicular distance between the movement trajectory and a virtual line connecting home position, movement endpoint, and movement extent (16).

Perceptual reports obtained via the pointer method provided an angular error, defined as the angle between reported and actual hand position, both relative to the home position, as well as a radial error, defined as the difference in radial distance of the reported versus actual hand position. The bias in perceived hand position was computed by averaging angular errors across localizations (after correcting for direction of rotation in *experiment 1* by reversing the sign of angular errors in cycles with a CW rotation). The variability of hand localization reports (inverse precision) was computed using the interquartile range (IQR) of angular errors (after subtracting the bias for each target in *experiment 1* separately). We chose IQR as a measure of variability due to its robustness to outliers.

Perceptual reports obtained via the 2AFC method were used to compute psychometric curves at the single-subject level. This was achieved by fitting a generalized linear regression model of the reported hand positions relative to the probe stimulus offset, for each cycle type (adaptation, washout) separately, using the glmfit.m function and a logit link function. Hand position reports were assumed to be binomially distributed. From the psychometric curves, biases in perceived hand position were computed as the point of subjective equivalence (PSE), i.e., as the (interpolated) probe stimulus offset at which subjects would be expected to report that the probe stimulus was “left” of their actual hand position in 50% of trials. Variability of hand localization reports was computed as the just noticeable difference (JND), i.e., the difference between the (interpolated) probe stimulus offsets at which subjects would be expected to report that the probe stimulus was “left” of their actual hand position in 75 versus 25% of trials. For computation of the JND in *experiment 1*, curves for each target were first computed separately. The data were then combined by first subtracting the respective PSE from the probe stimulus offsets for each target, thereby effectively shifting the psychometric curves along the *x*-axis to align at x = 0. A joint psychometric curve was then computed based on the x-shifted data points from both targets (as shown in Fig. 3*C*).

We rejected kinematic data of reaches that were too slow (movement duration > 300 ms to reach target radius) or had a strong curvature, indicated by LI > 0.2. In *experiment 1*, where two targets were presented, reaches toward the wrong target were excluded (movement direction < −30°). During pointer localization, participants occasionally pressed the foot pedal prematurely by accident. Therefore, hand position reports with high radial errors were rejected (defined as radial errors of hand position report that were less than half the actual movement extent, or radial errors that exceeded a threshold of two times the standard deviation of all radial errors).

Kinematic data were computed separately for adaptation and washout cycles and binned depending on the reach number in a cycle. For example, the adaptation aftereffect was computed as the mean movement direction during the first reach across all washout cycles. For all analyses of kinematics, we included reaches with and without visual feedback, i.e., also reaches in localization trials, as we expected no difference between these trials regarding the kinematics of ballistic reaching movement. Similarly, hand localization data derived from the pointer method in *experiments 1* and *2* were binned depending on cycle length. Thus, we were able to compute hand localization bias and variability for different time points, i.e., after two, three, or four movements, during adaptation and washout. This allowed us to capture any short-term dynamics of perceived hand position.

### Statistics

Statistics were computed in MATLAB R2020b and JASP 0.14.0.0 (17). We reported mean values and standard deviation or, when a Shapiro-Wilk test indicated a violation of the assumption of normality, median, and interquartile range. Mixed analyses of variance (ANOVA) were used to test for differences in movement kinematics between different conditions and between participants of different groups. Perceptual variables obtained from pointer localizations were submitted to repeated-measures ANOVA. Perceptual variables from 2AFC localizations were analyzed using paired-sample *t* tests. When the assumption of normality was violated, we used a corresponding nonparametric test. *t* test was one-sided when we tested for effects previously reported in the literature and two-sided when effects were not known a priori from the previous literature. We used mixed and repeated ANOVA, as well as paired-sample *t* tests for additional post hoc analysis. Bayesian *t* tests were used where the goal was to test for evidence in favor of the null hypothesis.

## RESULTS

### *Experiment 1*: Reaiming Task

In the first experiment, our primary hypothesis was that the precision of perceived hand position decreases during early adaptation to a visuomotor rotation. We expected adaptation to be evident in a gradual overcompensation of movement direction (4), i.e., an increase in cursor error from the first to the fourth reach during adaptation, accompanied by a bias in hand localization toward the rotated visual feedback.

#### Adaptation of reaching movements.

**Figure 2.**
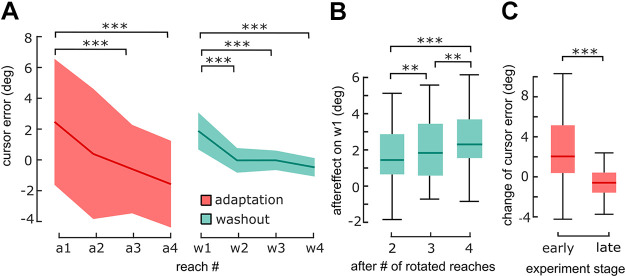
Adaptation of reaching movements in *experiment 1*. *A*: median cursor error (bold lines, ± semi-IQR indicated by shading, *n* = 30 participants, collapsed across the two groups of *experiment 1*) was shifted in opposite direction relative to the rotated visual feedback (±45° VMR) across consecutive reaches of adaptation cycles (red, “a,” numbers represent order of reaches, e.g. “a1” being the first reach of adaptation cycles, etc.). The first reach of washout cycles (cyan, “w1”) showed an adaptation aftereffect, which decreased over consecutive movements (“w2,” etc.). *B*: implicit motor adaptation was evident as an increase in aftereffects (boxplots, bold lines shows median) with the number of preceding reaches performed with rotated visual feedback. *C*: change of cursor error from first to second reach in adaptation cycles was positive during the first 30 cycles of the experiment (*left*, boxplot) but negative during the last 30 cycles (*right*). Positive values reflect overcompensation. Negative values reflect adjustment of aiming strategy. **P < 0.01, ***P < 0.001 (*A* and *B* show post hoc *t* tests, *C* shows rANOVA interaction term). IQR, interquartile range.

We found small cursor errors (angular error between cursor and cursor target) during adaptation cycles, indicating that participants successfully applied the instructed reaiming strategy to compensate for the visuomotor rotation. Instead of the expected gradual overcompensation (4), we found that cursor error was significantly greater than zero during the first reach of adaptation cycles [a1: 2.47 ± 7.81° (median ± IQR), W = 359.0, *P* = 0.008, *r* = 0.54, test against 0] and decreased across subsequent adaptation reaches (Fig. 2*A*). We performed a 2 × 4 × 2 mixed-effects ANOVA to test for effects of the within-subject factors cycle type (adaptation and washout) and reach number (i.e., 1st reach, 2nd reach, 3rd reach, and 4th reach in a cycle) on cursor error. In addition, we included the between-subjects factor group (pointer group and 2AFC group). We found a main effect for factors cycle type [*F*(1,28) = 4.49, *P* = 0.04, η^2^ = 0.06] and reach number [*F*(3,84) = 39.98, *P* < 0.001, η^2^ = 0.02), and interaction between cycle type and reach number [*F*(3,84) = 4.79, *P* = 0.004, η^2^ = 0.003]. Post hoc tests showed a significant decrease in cursor error from the first to the following reaches of washout [all *t*(29) > 5.90, all *P* < 0.001 for 1st vs. 2nd, 1st vs. 3rd, and 1st vs. 4th, Holm correction], as shown from w1 to w4 in Fig. 2*A*. Cursor error decreased also across adaptation cycles [*t*(29) = 4.46 for 1st vs. 3rd, *t*(29) = 6.09 for 1st vs. 4th, both *P* < 0.001, Holm correction]. Adaptation between both groups did not differ significantly (all *P* values for interactions with the factor group > 0.1). We therefore collapsed data across the two groups for further kinematic analysis.

Although the decrease of cursor error during adaptation cycles is inconsistent with the overcompensation reported as evidence of implicit learning during instructed strategy use in previous studies (4), we found aftereffects during washout cycles, which are considered a hallmark of implicit adaptation (see w1 in Fig. 2*A*). Furthermore, aftereffects increased with the number of immediately preceding rotated reaches [*F*(2,58) = 22.17, *P* < 0.001, η^2^ = 0.43, Fig. 2*B*; *t*(29) = −3.19, *P* = 0.002, two vs. three preceding reaches; *t*(29) = −3.47, *P* = 0.002, three vs. four preceding reaches; Holm correction], providing evidence of gradual implicit learning throughout adaptation cycles.

We compared the dynamics of cursor error during adaptation between early and late stages of the experiment (first vs. last 30 adaptation cycles). A 2 × 2 rANOVA with the within-subject factors reach number (1st reach and 2nd reach) and experiment stage (early and late) showed no effect of reach number [*F*(1,29) = 2.55, *P* = 0.12], but a main effect of experiment stage [*F*(1,29) = 6.09, *P* = 0.02, η^2^ = 0.15] and interaction between reach number and experiment stage [*F*(1,29) = 19.28, *P* < 0.001, η^2^ = 0.04, see Fig. 2*C*]. This indicates that subjects did not simply use the instructed strategy, but adjusted it throughout the experiment via ongoing strategy-based learning. Post hoc tests showed that cursor error increased from the first (5.05 ± 8.43°) to the second reach of adaptation cycles early during the experiment [7.34 ± 9.93°, *t*(29) = 3.68, *P* = 0.004, d = 0.7, Holm correction], consistent with gradual overcompensation. However, at later stages of the experiment, cursor error decreased from first (3.41 ± 8.12°) to the second reach of adaptation cycles (2.27 ± 7.47°, W = 363.0, *P* = 0.01, *r* = 0.56, Holm correction), consistent with the idea that subjects adjusted the instructed aiming strategy across the course of the experiment (see also Supplemental Analysis S1; all Supplemental material is available at https://doi.org/10.5281/zenodo.11084801).

### Changes in Hand Localization

**Figure 3.**
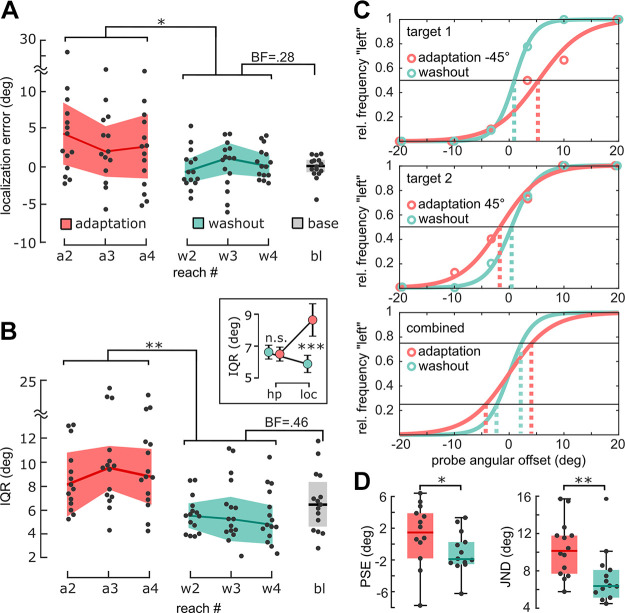
Changes in hand localization in *experiment 1*. *A*: median hand position reports for reaches during adaptation (red bold line, ± semi-IQR indicated by shading), washout (cyan bold line), and baseline (black line). Hand localization was biased toward the rotated cursor (±45° VMR) in adaptation cycles, compared with washout cycles. Numbers indicate the reach number in a cycle at which participants reported their hand position, e.g. “a2” after the second movement in an adaptation cycle (the first reach never required localization). *B*: interquartile range of hand localization errors (IQR, bold lines show median, ±semi-IQR indicated by shading) was increased during adaptation cycles, compared with washout cycles across all reach numbers. The *inset* shows localization variability (loc) when hand position variability (hp) was matched between cycle types. *C*: example psychometric curves (solid lines) fitted to 2AFC reports (circles) for target 1 (*top*) and target 2 (*middle*). For each participant in the 2AFC group, the point of subjective equivalence (PSE) was computed for both targets (dashed lines, note different direction of localization bias for different rotation directions), and subsequently subtracted from the respective probe angular offset values. The unbiased probes for both targets/rotation directions were then combined to compute the just-noticeable-difference (JND, *bottom*, interval between dashed lines). *D*: PSEs were biased in adaptation, compared with washout cycles (*left*, bold lines show median). JND was increased in adaptation, compared with washout cycles (*right*). *P < 0.05, **P < 0.01, ***P < 0.001; *n* = 15 participants for *A* and *B*, and *n* = 14 participants for *D, A* and *B* show rANOVA main effect of cycle type (adaptation and washout) and Bayesian *t* test between washout and baseline, *C* shows paired *t* tests. 2AFC, two-alternative forced choice; IQR, interquartile range.

As expected, we found that perceived hand position was biased toward the rotated cursor during adaptation cycles for both the pointer and the 2AFC groups. For the pointer group, angular hand localization errors during adaptation and washout cycles were 1.96 ± 6.18° and 0.46 ± 3.05° (median ± IQR), respectively [*F*(1,14) = 4.63, *P* = 0.049, η^2^ = 0.23; Fig. 3*A*, detailed statistics in Supplemental Analysis S2). In the 2AFC group^1^, localization bias was 1.69 ± 3.55° during adaptation, and −0.74 ± 4.38° during washout cycles [*t*(13) = 2.27, *P* = 0.02, d = 0.61, one sided; Fig. 3*D*].

Importantly, in both groups, we also found an increase in interquartile range (IQR) of angular localization errors during adaptation cycles, compared with washout cycles. For the pointer group, the IQR during adaptation cycles was 10.58 ± 4.98°, whereas IQR for washout cycles was 6.02 ± 1.83°, i.e., comparable to an IQR of 6.78 ± 2.48° during baseline (BF_10_ = 0.46). Thus, we compared precision during adaptation cycles to washout cycles and omitted the (time-consuming) baseline measurement in all subsequent experiments. We conducted a 2 × 3 rANOVA with the within-subject factors cycle type and reach number and found an effect of cycle type [*F*(1,14) = 15.67, *P* = 0.001,η^2^ = 0.36, Fig. 3*B*]. There was no significant effect of reach number [*F*(2,28) = 0.58, *P* = 0.57], nor an interaction between both factors [*F*(2,28) = 0.06, *P* = 0.95]. For the 2AFC group, the average JND was 10.41 ± 3.07° for adaptation and 6.87 ± 3.45° for washout. A paired *t* test in this group showed that the JND differed significantly between the two cycle types [*t*(13) = 3.99, *P* = 0.002, d = 1.07, Fig. 3*D*].

It is possible that participants based their estimate of hand location (partly) on the remembered location of the target. If the difference between the actual hand position and the remembered visual target exhibited higher variability during adaptation cycles, compared with washout cycles, any such bias in hand localization toward the target could have inflated variability in hand perception in adaptation cycles. Indeed, we found evidence that hand positions became systematically more variable during adaptation (11.32 ± 5.67°), compared with washout [3.80 ± 0.64°, *t*(28) = 7.08, *P* < 0.001, d = 1.32]. However, we could rule out the possibility that the observed increase in hand localization variability during adaptation merely reflected an increase in actual hand position variability. Specifically, we conducted a control analysis that matched hand position variability during localizations between adaptation and washout cycles in the pointer group. This was done by iteratively removing trials with extreme hand positions in adaptation, and close-to-average hand positions in washout, until hand position variability during adaptation was just below that during washout cycles [85 trials removed on average across subjects (median; range 48–127), 66 trials remaining (median; range 32–84)]. We then recomputed the variability of hand localization for the remaining trials and performed a 2 × 2 rANOVA with the within-subject factors cycle type (adaptation or washout) and type of variability (hand position or localization). We found a main effect of cycle type [*F*(1,14) = 11.3, *P* = 0.005, η^2^ = 0.13], and, importantly, an interaction between cycle type and type of variability [*F*(1,14) = 12.7, *P* = 0.003, η^2^ = 0.16, see Fig. 3*B,* *inset*]. Confirming that our matching procedure was successful, post hoc *t* tests showed that hand position variability was not significantly greater in adaptation compared with washout cycles [adaptation: 6.51 ± 1.56°, washout: 6.63 ± 1.65°, *t*(14) = 0.2, *P* = 0.88, Holm correction; BF_10_ = 0.09, one-sided]. However, hand localization variability was still higher in adaptation, compared with washout [adaptation: 8.61 ± 3.92°, washout: 5.90 ± 2.14°, *t*(14) = 4.90, *P* < 0.001, d = 1.08, Holm correction, BF_10_ = 12.52]. We performed a further control analysis to exclude the possibility that the observed difference in hand localization variability could be explained by a systematic drift in localization bias across the experiment (see Supplemental Analysis S3).

### *Experiment 2*: Aim-Direct Task

We next asked whether the observed increase in hand localization variability during adaptation was limited to conditions under which subjects use reaiming strategies, or exists more generally even when learning is purely implicit. In *experiment 2*, we thus instructed participants to ignore the rotation of the cursor relative to movement direction during adaptation cycles and to aim directly at the target. Due to the instruction to move the hand through the target, we report the movement direction, rather than the cursor error as in *experiment 1*.

#### Adaptation of reaching movements.

**Figure 4.**
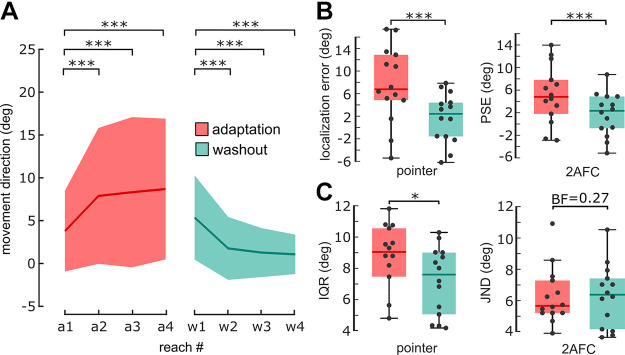
Kinematic and perceptual results in *experiment 2*. *A*: mean movement direction for each reach during adaptation (red bold line; reaches a1–a4) and washout (cyan bold line; reaches w1–w4; *n* = 33 participants, across groups). Shadings indicate std. Movement direction was shifted in the direction opposite to the rotated visual feedback (45° VMR) for all reaches during adaptation cycles (“a1” being the first reach in an adaptation cycle, “a2” the second reach in an adaptation cycle, etc.). The first reach in a washout cycle (“w1”) showed an adaptation aftereffect, which decreased over consecutive movements (“w2,” etc.). *B*: hand position reports (boxplots, dots represent single participants, bold line shows median) were shifted in the direction of visual feedback during adaptation (red) compared with washout cycles (cyan) for pointer (*left*, *n* = 14) and 2AFC (*right*, *n* = 14) groups. *C*: variability increased in adaptation, compared with washout, for the pointer group (*left*). However, we did not find any difference between the two cycle types for the 2AFC group (*right*). *P < 0.05, ***P < 0.001, *A* shows post-hoc *t* tests, *B* and *C* show a rANOVA main effect of cycle type for the pointer group (*left*, averaged over factor reach number, which was not significant) and paired *t* tests for the 2AFC group (*right*). 2AFC, two-alternative forced choice.

We performed a 2 × 4 × 2 mixed ANOVA with the within-subject factors cycle type (adaptation and washout) and reach number (i.e., 1st reach, 2nd reach, 3rd reach, and 4th reach in a cycle). In addition, we included the between-subjects factor group (pointer group and 2AFC group) to confirm that the localization method had no effect on movement adaptation. We found a main effect for factors cycle type [*F*(1,31) = 65.93, *P* < 0.001, η^2^ = 0.23, Fig. 4*A*) and an interaction between cycle type and reach number [*F*(3,93) = 76.23, *P* < 0.001, η^2^ = 0.11]. Movement direction gradually shifted in CCW direction from the first to the fourth movement during adaptation cycles [all *t*(28) < −7.84, all *P* < 0.001 for 1st vs. 2nd, 1st vs. 3rd, and 1st vs. 4th, *t*(28) = −2.85, *P* = 0.03 for 2nd vs. 3rd, Holm correction] and reverted toward zero during washout cycles [all *t*(28) > 7.74, all *P* < 0.001 for 1st vs. 2nd, 1st vs. 3rd, and 1st vs. 4th, Holm correction]. Adaptation between both groups did not differ significantly (all *P* values for interactions involving group >0.6). Changes in movement direction were much smaller than the visuomotor rotation, so that the cursor during adaptation cycles obviously missed the target, indicating that participants did not apply a compensatory movement strategy during adaptation cycles and that motor learning was implicit.

#### Changes in hand localization.

As in *experiment 1*, we found a bias in hand localization in the direction of the rotated cursor during adaptation cycles for both groups^2^. Examining angular localization errors as a function of reach number in a cycle in the pointer group, we found that the perceived hand position was biased toward the cursor after a single movement with rotated visual feedback (see Fig. 4*B*, for statistical analysis see Supplemental Analysis S2).

To test if IQR of angular localization errors was again higher during adaptation, compared with washout, we performed a 2 × 3 rANOVA for the pointer group with within-subject factors cycle type (adaptation or washout) and reach number (i.e., 2nd, 3rd, or 4th reach in a cycle). This revealed a significant effect of cycle type, with higher hand localization IQR during adaptation (8.83 ± 1.93°), compared with washout [7.50 ± 2.05°; *F*(1,14) = 6.34, *P* = 0.03, η^2^ = 0.15, Fig. 4*C*]. We did not find a significant effect of reach number [*F*(2,28) = 1.09, *P* = 0.35], nor an interaction between both factors [*F*(2,28) = 0.86, *P* = 0.43]. However, for the 2AFC group, we did not find a significant difference between angular localization JND during adaptation and washout cycles [*t*(13) = 0.02, *P* = 0.99]. We performed an additional Bayesian *t* test and found moderate evidence in favor of the null hypothesis, i.e., that JNDs in both cycle types were indeed not different (BF_10_ = 0.27). This discrepancy between pointer and 2AFC methods may result from a smaller overall effect compared with *experiment 1* (difference in localization variability between adaptation and washout cycles *exp. 1*: 4.06 ± 3.79°, *exp. 2*: pointer only: 1.33 ± 2.03°, W = 293.0, *P* = 0.019, *r* = 0.44). Given that any difference between adaptation and washout, if truly present, was smaller in *experiment 2*, compared with *experiment 1*, it was a priori more likely to miss any true difference in localization variability between adaptation and washout. We performed a control analysis to exclude the possibility that differences in target position across experiments explain the smaller effect in *experiment 2* (see Supplemental Analysis S4). We can only speculate if other factors, such as differences in sensitivity between localization methods, contributed to differences in the dynamics of localization variability between the groups.

### *Experiment 3*: Cued Localization

In *experiment 3*, we tested whether the decrease in hand localization precision observed in *experiment 1* persisted even when participants could allocate attention for hand localization. To this end, participants completed a close variant of *experiment 1*; however, hand localizations were precued, and therefore predictable. As we speculated in *experiment 2* that the 2AFC method may be less sensitive to smaller differences in hand localization variability, *experiment 3* used the pointer method only.

#### Movement adaptation.

**Figure 5.**
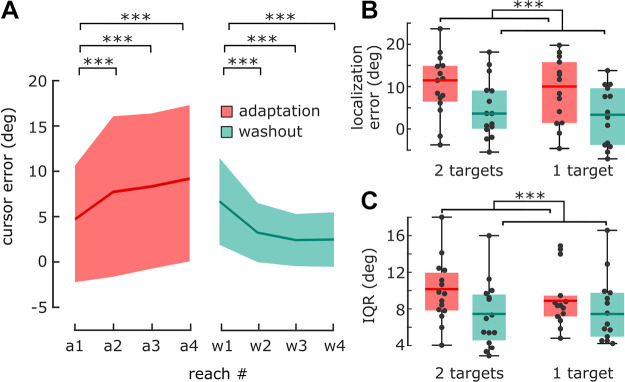
Kinematic and perceptual results in *experiment 3*. *A*: mean movement direction for each reach during adaptation (red bold line; reaches a1–a4, shading indicates std.) and washout (cyan bold line; reaches w1–w4; *n* = 28 participants, across groups). Movement direction was shifted in the direction opposite to the rotated visual feedback (45° VMR) for all reaches during adaptation cycles (“a1” being the first reach of adaptation cycles, “a2” the second reach of adaptation cycles, etc.). The first reach of washout cycles (“w1”) showed an adaptation aftereffect, which decreased over consecutive movements (“w2,” etc.). *B*: hand position reports (boxplots, dots represent individual participants, bold lines show median) were shifted in the direction of visual feedback during adaptation, compared with washout, for two-target (*left*, *n* = 14 participants) and one-target (*right*, *n* = 14 participants) groups. *C*: variability increased in adaptation, compared with washout, for the two-target (*left*) and one-target groups (*right*). ***P < 0.001, *A* shows post hoc *t* tests, *B* and *C* show a rANOVA main effect of cycle type.

We performed a 2 × 4 × 2 mixed ANOVA with the within-subject factors cycle type (adaptation or washout) and reach number (i.e., 1st reach, 2nd reach, 3rd reach, and 4th reach in a cycle) and between-subject factor group (2-target or 1-target). We found a significant effect of cycle type [*F*(1,27) = 13.41, *P* = 0.001, η^2^ = 0.08, Fig. 5*A*] and an interaction between cycle type and reach number [*F*(3,81) = 43.64, *P* < 0.001, η^2^ = 0.07). The interaction was due to a gradual increase of cursor error from the first to the fourth reach during adaptation cycles [1st vs. 2nd, 1st vs. 3rd, and 1st vs. 4th, all *t*(28) < −5.68, all *P* < 0.001, Holm correction, a1 to a4 in Fig. 5*A*] and a decrease of cursor error during washout cycles [1st vs. 2nd, 1st vs. 3rd, and 1st vs. 4th, all *t*(29) > 5.01, all *P* < 0.001, Holm correction, w1 to w4 in Fig. 5*A*]. As in *experiment 1*, participants used a compensatory aiming strategy during adaptation in these experiments. Unlike *experiment 1*, *experiment 3* did not reveal any adjustment of the aiming strategy across the experiment [change of cursor error from 1st to 2nd reach in adaptation, first 30 cycles: 3.30 ± 4.05°, last 30 cycles: 2.97 ± 4.88°, *t*(27) = 0.47, *P* = 0.64, BF_10_ = 0.22]. This is consistent with the observation that participants in *experiment 1* adjusted their aiming strategy only at later stages of *experiment 1*. *Experiment 3* had fewer blocks than *experiment 1*, possibly preventing the later-stage strategy adjustment that we observed in *experiment 1*. There was neither an effect of group, nor an interaction of group with either of the two within-subject factors (all *P* > 0.1).

#### Changes in hand localization.

As in previous experiments, angular hand localization errors were biased toward the rotated cursor in adaptation cycles, compared with washout cycles (see Fig. 5*B*, for statistical analysis see Supplemental Analysis S2).

A 2 × 2 mixed ANOVA of hand localization variability with the within-subject factor cycle type, and the between-subject factor group, revealed a significant effect of cycle type [adaptation: 9.50 ± 3.28°, washout: 7.61 ± 3.52°, *F*(1,27) = 18.83, *P* < 0.001, η^2^ = 0.07, Fig. 5*C*], but no effect of group [*F*(1,27) = 0.01, *P* = 0.92], nor an interaction between cycle type and group [*F*(1,27) = 2.50, *P* = 0.13]. Consistent with results from *experiment 1*, hand localization reports were thus more variable in adaptation cycles, compared with washout cycles. However, this variability increase (1.89 ± 2.38°) was smaller than in *experiment 1* [4.06 ± 3.79°, W = 552.0, *P* = 0.01, *r* = 0.31]. In summary, we show that the decrease of localization precision observed in *experiment 1* is only partly resistant to an allocation of attention to hand localization and that attention seems to play a role. This is supported by further evidence that attention to vision can decrease localization precision, which we report in Supplemental Experiment S6.

## DISCUSSION

We report that the precision with which subjects can localize their hand changes during early motor adaptation. Our results show that precision of hand localization decreases rapidly once a visuomotor rotation is introduced in a reaching task. This decrease is observed when subjects use a compensatory aiming strategy (*experiment 1*) and persists even when subjects can allocate attention to hand position (*experiment 3*), and, at least under certain circumstances, when learning is purely implicit (*experiment 2*). Position sense in general, and its precision in particular, are increasingly recognized as important determinants of motor adaptation (2, 8). Hence, the precision decrease observed during early adaptation cycles in our study may have important implications for our understanding of how movements are adapted.

We discuss how the observed dynamics in hand localization could relate to mental processes associated with implicit or strategy-based learning, outline potential consequences of changes in the precision of hand localization for motor learning, and finally discuss the limitations of our study, together with potential future directions.

### Component Processes of Adaptation That May Impact on Localization Precision

We observed the largest decrease in hand localization precision in *experiment 1*, when movements were governed both by strategy-based learning and implicit adaptation. *Experiment 2* revealed that this decrease can persist in the absence of any reaiming strategy, when adaptation is purely implicit, however, this decrease was significantly smaller than in *experiment 1*. This supports the idea that hand localization becomes less precise during adaptation in particular when cognitive strategies are involved.

Cognitive reaiming strategies in visuomotor adaptation likely shift attention from the hand to the (rotated) visual feedback. Such intermodal attention results in a gain modulation that down-weights somatosensory information (18). We therefore speculate that one reason why hand localization becomes less precise during adaptation could be an intermodal shift of attention during strategy use.

Consistent with this idea, hand localization variability increased less in *experiment 3*, compared with *experiment 1*. As we have shown, participants in *experiment 1* adjusted the instructed strategy throughout the experiment, in line with previous reports (e.g., 14). It seems plausible that this continuous strategy adjustment caused uncertainty in the aiming location and involved careful monitoring of the relation between (rotated) visual feedback and cursor target, i.e., attention to vision, rather than proprioception. In the localization trials of *experiment 3*, on the other, participants could focus on hand localization, rather than shifting attention to vision, and aim directly at the visual target. This may explain why the increase in variability of hand localization was less pronounced in *experiment 3*. Interestingly, a significant increase in variability nevertheless persisted in *experiment 3*. This indicates that the recent history of strategy use resulted in an enduring decrease of precision even in localization trials, when subjects could attend to hand position. The observed decrease in precision is therefore not merely a short-lived result of momentary inattention.

*Experiment 2* showed that the observed decrease in precision can be resistant to aiming directly at the target. However, while participants were instructed to ignore the cursor, attention may nevertheless play a role for the observed decrease in precision. Vision typically conveys essential information about limb positions, in particular in relation to the external world. It may be therefore difficult to ignore visual feedback completely. For example, visual feedback that is invariant to movement direction (also referred to as error-clamped feedback) drives feedforward motor adaptation even though participants are made aware of the manipulation and instructed to ignore the visual feedback (19, 20). We present further evidence that supports a role of attention in the precision decrease in Supplemental Experiment S6.

We observed a pronounced localization precision loss when participants used an aiming strategy (in *experiment 1*). Strategic reaiming is driven by cursor error (or performance error), i.e., the error between visual feedback and the intended target (14, 21). Could cursor error explain the decrease in precision? If so, we would expect to find a similar decrease in precision after the first reach of washout, where participants experienced cursor errors due to the aftereffect, which were comparable in magnitude to cursor errors during adaptation cycles (see Fig. 2*A*). However, precision returned to baseline levels already in the second washout trial (Fig. 3*A*). Furthermore, participants experienced larger cursor errors during the early stage of *experiment 1*, compared with the late stage (where they adjusted their aiming strategy, presumably based on the cursor errors they had experienced before, thus reducing error). If cursor error played a role for localization precision, we would expect to find a more pronounced precision loss during the early stage of *experiment 1*, which was not the case (see Supplemental Analysis S3).

Besides attention, another factor that may contribute to the decrease in precision is the prediction error that a visuomotor rotation necessarily introduces. The cerebellum plays a role in position sense during active movements (22, 23). Predictive computations of the cerebellum may enhance the precision of position sense (24). Similar to integration of multiple sensory modalities, combination of sensory and predictive information may depend on their respective uncertainty (25). However, when predictive models are updated to a change in visuomotor contingency, their estimation uncertainty is thought to increase due to the presence of prediction errors, which are most pronounced in the initial stage of motor adaptation (26). Therefore, another possible explanation for the decrease in precision of hand localization during early visuomotor learning in our study is a reduction in the precision of predictive information. However, if prediction error played a major role in the observed decrease in hand localization precision, we would have expected to observe a similar decrease in *experiments 1* and *2*, as prediction errors should have been similar across experiments (same rotation magnitude). Furthermore, we would have expected to find a decrease in washout cycles, in which participants experienced errors between predicted and perceived movement outcomes as well, evident in adaptation aftereffects (albeit likely of substantially smaller size). This was not the case, nor did we find a deterioration of perceptual precision between the baseline condition and washout cycles in *experiment 1*.

### Dynamics of Perceptual Changes during Visuomotor Adaptation

Previous work on the neural processing of somatosensory feedback during visuomotor adaptation suggests suppression of proprioceptive information during the early period of learning (27–29). However, these neurophysiological studies have left the question unanswered whether the precision of perception changes. The few psychophysical studies that have investigated variability of position sense in a context of visuomotor adaptation have done so at a late stage of adaptation and found no decrease in precision (7, 30).

This may not be surprising, given that a decrease in precision is more likely during the early period of motor learning, when reaiming strategies dominate learning. Indeed, we found a decrease that was already present after a single reaching movement with rotated visual feedback. Precision recovered immediately and completely (compared with baseline) during washout cycles. Importantly, hand localization trials were identical across both types of cycles. That is, the reaching movements during these trials were performed in the absence of visual feedback, both in adaptation and washout cycles. Precision of hand localization therefore did not depend on the immediate visual feedback in the current trial, but likely on the focus of attention directed by the task requirements during the present reach (*experiment 1*) and in recent trials (*experiment 3*). Furthermore, precision did not change across the three reaching movements of an adaptation cycle. However, we expect precision of hand localization to recover over a longer course of visuomotor adaptation, as previous studies reported that variability of position sense during late stages of adaptation is not different from baseline (7, 30). This could be because participants likely rely less on visual attention during late stages of adaptation when a successful reaiming strategy has already been established (31).

In addition to a decrease in hand localization precision, we observed that the visuomotor rotation introduced a systematic bias in localization, consistent with recalibration of proprioception. Cross-sensory recalibration has been regarded as a gradual process (32, 33). Here, we observed a notably fast localization bias, occurring even after a single reaching movement with rotated visual feedback. This finding confirms previous studies, which have reported similarly fast shifts for proprioception (34, 35). Importantly, perceptual changes did not increase over the observed period of reaches, speaking against the idea of a gradual change. Hand localization was performed in the absence of visual feedback and should thus reflect recalibration of proprioception, and not multisensory integration, as the latter requires simultaneous input from two sensory modalities.

### Potential Consequences of a Decrease in Hand Localization Precision for Motor Learning

A recent model casts implicit motor adaptation as the result of an error between desired and sensed hand position, where the latter is derived from a proprioceptive estimate (2). During visuomotor rotation, proprioception is shifted toward visual feedback due to sensory recalibration and partial multisensory integration (36). The rules of multisensory integration are governed by the precision of each sensory estimate, such that less precise estimates are shifted more strongly (36). Reduced proprioceptive precision during visuomotor adaptation should thus result in a stronger shift of the estimated hand position toward visual feedback due to (partial) multisensory integration. As a consequence, following the idea that a shift in proprioception contributes to implicit adaptation, a reduction in proprioceptive precision during visuomotor adaptation should enhance implicit adaptation. Thus, the dynamic change of precision in hand localization observed here might act as a mechanism that controls the speed of adaptation.

We found that the precision of hand localization was lowest in *experiment 1*, when participants used a compensatory aiming strategy. Thus, we would expect strategic reaiming to enhance implicit adaptation. This is because lower precision has been associated with stronger implicit motor adaptation (2, 8). However, comparing *experiment 1*, where participants used a movement strategy, and *experiment 2*, where participants were instructed to aim directly at the movement target, we found that aftereffects, as indicators of implicit adaptation, were stronger when adaptation was purely implicit, i.e., in *experiment 2* (see Supplemental Analysis S5; however, note that, in contrast to *experiment 2*, *experiment 1* used an experimental design with alternating directions of the visuomotor rotation, which may account for these differences in aftereffect.). This finding is in line with several previous studies that report a negative relationship between implicit adaptation and strategic reaiming (37, 38). The relationship between precision of hand localization and implicit adaptation may thus depend on the presence of a reaiming strategy.

However, implicit adaptation likely depends not solely on changes to proprioception. Patients with cerebellar ataxia exhibit reduced proprioceptive precision compared with healthy controls (22–24), however, they adapt less than controls. Both the reduction in adaptation, and in proprioceptive precision, are attributed to an impairment of internal predictive models in these patients. The perception of limb position, and its relationship to implicit motor adaptation, therefore appear to be more complex, and require further studies.

### Limitations and Future Directions

Our study was partly motivated by recent reports that highlight the role of proprioception in implicit motor adaptation (2). However, our sense of limb position is complex and reflects various sources of information, including expectation about the intended action, efferent information, proprioception, and vision (39). Here, participants reported hand positions after active movements, blending information from multiple sensory sources. To assess the influence of intermodal attention on proprioception specifically, future studies may include a condition in which hand localization follows passive movements.

We consistently observed a decrease in hand localization precision when participants used a reaiming strategy to reduce movement errors (*experiments 1* and *3*). The use of compensatory strategies has been linked to eye movements toward the aim point before movement onset (10, 11). We speculate that participants may have changed their gaze location from the hand target toward the cursor target after movement initiation to perceive the cursor error. An associated shift in spatial attention may be critical for the decrease of localization precision, as precision decreased less when participants were instructed to ignore the cursor (*experiment 2*). However, we were not able to control for this shift. Future studies could use gaze tracking to examine the influence of eye movements on localization precision.

To assess variability of perceived hand position as a proxy of proprioceptive precision, we would ideally examine hand localization variability during a single, extended adaptation period of many trials, followed by an extended washout period, i.e., using a classic design of motor adaptation studies. Computing variability requires repeated measurements. However, during early motor learning, movements and perception change rapidly (34, 35). To circumvent this nonstationarity, which could potentially inflate perceptual variability, we decided to probe perception once during early adaptation and repeat this early adaptation stage many times. However, adaptation may change with repetition, and thus differentially affect perceptual measures across the course of the experiment.

### Conclusions

We report for the first time that the precision of hand localization decreases during early adaptation to a perturbation of visual movement feedback. This reduction may be caused by attention to visual versus proprioceptive information during strategy-based motor adaptation.

## DATA AVAILABILITY

All raw data is available under https://doi.org/10.5281/zenodo.11084801.

## SUPPLEMENTAL DATA

10.5281/zenodo.11084801 Supplemental Analysis S1–S6: https://doi.org/10.5281/zenodo.11084801.

## GRANTS

M.W. was supported by Otto-von-Guericke University Medical Faculty LOM scholarship. M-P.S. was supported by a VolkswagenStiftung Freigeist Fellowship, project-ID 92977, and received funding from a Deutsche Forschungsgemeinschaft Sonderforschungsbereich, SFB-1436, TPC03, project-ID 425899996.

## DISCLOSURES

No conflicts of interest, financial or otherwise, are declared by the authors.

## AUTHOR CONTRIBUTIONS

M.W. and M-P.S. conceived and designed research; M.W. performed experiments; M.W. analyzed data; M.W. and M-P.S. interpreted results of experiments; M.W. prepared figures; M.W. drafted manuscript; M.W. and M-P.S. edited and revised manuscript; M.W. and M-P.S. approved final version of manuscript.

## Footnotes

1 We excluded one subject from all analyses of hand localization data who did not follow instructions during the hand localization.

2 We excluded one subject of the pointer group from the analysis due to implausibly large angular localization errors during washout cycles. In the 2AFC group, the PSE of two participants could not be computed correctly during adaptation cycles as relative frequency of answering “left” for all probe values fell short of 0.5. Furthermore, the psychometric curves obtained for washout cycles of two subjects exhibited a very sharp slope, resulting in a JND that was implausible (<0.3°). Thus, we excluded these subjects from the analysis.
